# Lateralization of social signal brain processing correlates with the degree of social integration in a songbird

**DOI:** 10.1038/s41598-020-70946-7

**Published:** 2020-08-24

**Authors:** Hugo Cousillas, Laurence Henry, Isabelle George, Schedir Marchesseau, Martine Hausberger

**Affiliations:** Univ Rennes, Normandie Univ, CNRS, EthoS (Éthologie animale et humaine) - UMR 6552, 35000 Rennes, France

**Keywords:** Neuroscience, Auditory system, Cognitive neuroscience, Neuronal physiology, Social neuroscience

## Abstract

Group cohesion relies on the ability of its members to process social signals. Songbirds provide a unique model to investigate links between group functioning and brain processing of social acoustic signals. In the present study, we performed both behavioral observations of social relationships within a group of starlings and individual electrophysiological recordings of HVC neuronal activity during the broadcast of either familiar or unfamiliar individual songs. This allowed us to evaluate and compare preferred partnerships and individual electrophysiological profiles. The electrophysiological results revealed asymmetric neuronal activity in the HVC and higher responsiveness to familiar than to unfamiliar songs. However, most importantly, we found a correlation between strength of cerebral asymmetry and social integration in the group: the more preferred partners a bird had, the more its HVC neuronal activity was lateralized. Laterality is likely to give advantages in terms of survival. Our results suggest that these include social skill advantages. Better knowledge of links between social integration and lateralization of social signal processing should help understand why and how lateralization has evolved.

## Introduction

Group cohesion relies in particular on the perception and appropriate processing of signals from conspecifics, i.e. on social cognition^1,2^. The ability to identify group membership^3^, to process social familiarity^4^ and to recognize individual group members^5^ appears to be widespread among animals. Songbirds in particular are very good models for studying links between “social and vocal brains”^4^. Since its discovery by Nottebohm^6^, their brains’ «song system» keep revealing more and more relationships between communication skills and social interactions^7^. However, understanding how social cohesion, communication skills and brain processes are interrelated requires us to bridge the gap between group functioning and brain processes.

Social relationships enhance attention^8^, which is important for memory formation and perceptual tuning^9^. The mere presence of a singing adult model in a group of young naive starlings is not sufficient for them to develop all typical adult song features. Individual bonding is essential to trigger appropriate selective attention, i.e. focusing on specific group members or social models^4,10^. Attention is a key element in perceptual lateralization^11^ and loss of attention can lead to total disappearance of the species-specific lateralized responses observed in brain processing of social signals^12,13^. Evolution of social attentional skills may have been part of social evolution and as such may have been associated with the evolution of perceptual lateralization^12^. Authors have proposed several different, sometimes contradictory, hypotheses concerning the advantages of sensory lateralization^14,15^. However, researchers generally accept that lateralized individuals possess higher skills for a variety of tasks, especially dual tasks requiring them, for example, to divide their attention between searching for food and detecting a predator^16^. Some types of lateralization develop with experience^18,19^. For example, hens’ type of mothering can influence chicks’ lateralization, probably through attentional processes involving social interactions with mother or between chicks^20^. Lateralization of zebra finches’ song processing in the secondary auditory area (NCM) correlates with the fidelity of their imitation of their model^21^. Recently, several reports have shown in addition the importance of the influence of the timing of memory formation on the direction and strength of the lateralization of song processing in this area^2,22–24^. Yet, we still do not know how the quality of interactions between individuals can influence social signal brain processing and its lateralization, especially concerning population level asymmetries. Since more gregarious starlings show increased levels of opioids^25^ and since more socially integrated (i.e. with more bonding partners) ravens are less stressed physiologically^26^, social bonding could well play a major role in guiding neurophysiological functioning.

European starlings are open-ended learners and their social bonding is associated with vocal sharing^27^. This is also the case for other social species of songbirds^28^. Outside the breeding season, individual adult starlings associate with preferred, often same-sex and same-age partners with which they share parts of their song repertoire. Changes in group composition lead to new associations and rapid changes in song repertoires so that newly formed partnerships are associated with new patterns of vocal sharing^29^. While females generally form same-sex pairs^30^, males form small same-sex groups whose individuals differ in their degree of «social integration» (i.e. number of preferred partners). This degree of «social integration» is correlated with the degree of vocal «conformity» (i.e. proportion of shared structures in the vocal repertoire)^29^. This suggests individual variations in the levels of social motivation, tolerance and/or social attention.

If lateralization of sensory processes reflects attentional and social skills, we would expect that the more socially «engaged» male starlings would show higher levels of lateralization in brain processing of social signals. To test this hypothesis, we determined first the social relationships within a group of non-breeding captive starlings and then we recorded the HVC (as a proper name) electrophysiological activity of some of the males while they were listening to either familiar or unfamiliar male starling songs. HVC neurons of starlings not only respond to the bird’s own song (as in other species^31^) but also to individual whistles of conspecifics^13,32^. These responses show a right hemisphere dominance when the birds are awake, but not when they are anesthetized^33^. This reinforces the idea that processing complex social signals requires attention^34^.

**Figure 1:**
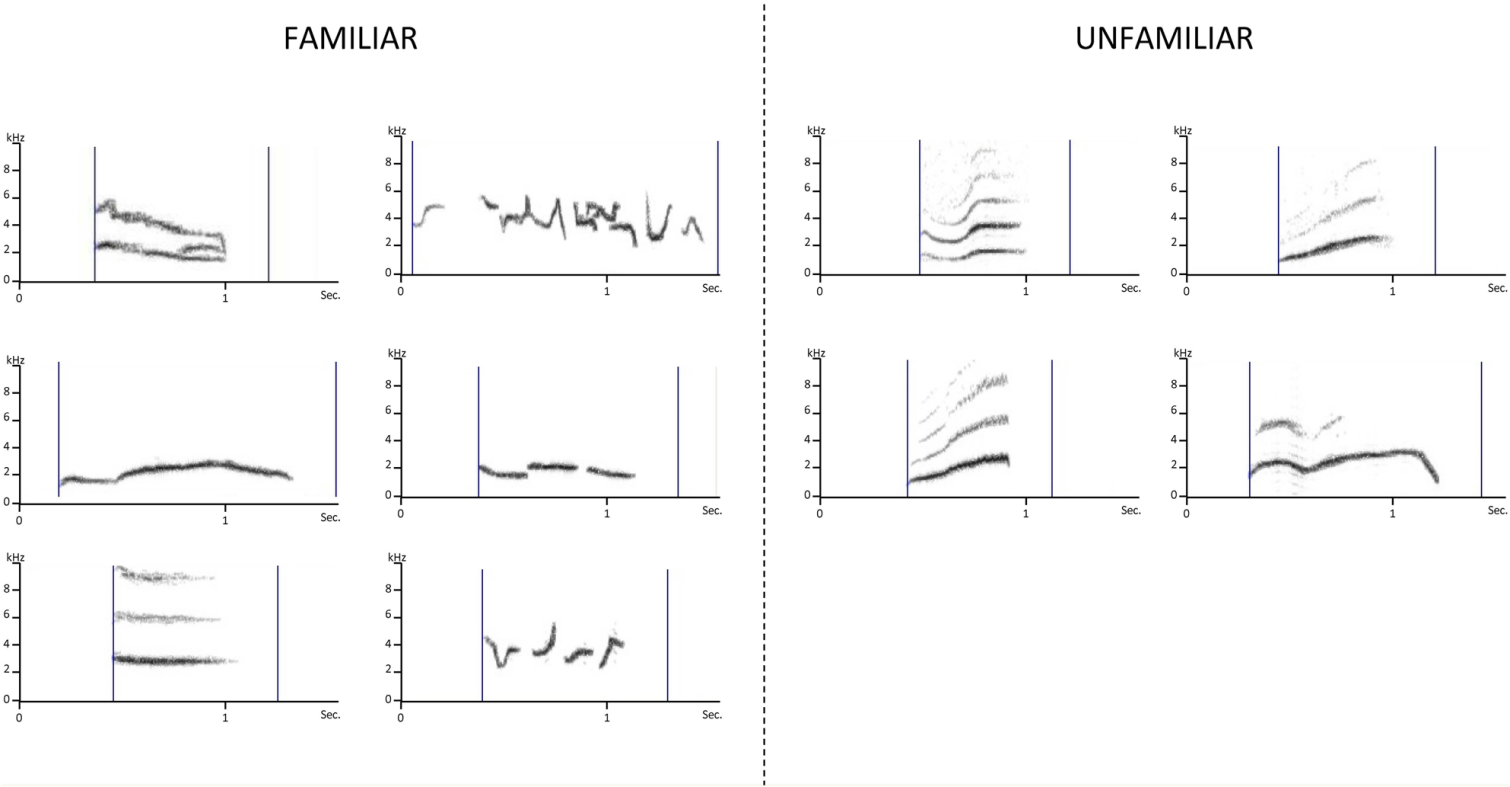
A set of stimuli including 10 Class-II individual male starling whistles. Left: six familiar whistles recorded in the experimental birds’ home aviary. These familiar whistles differed from one bird to another (birds’ own songs were not included). Right: four whistles recorded from unfamiliar distant birds. These unfamiliar whistles were the same for all birds.

Our subjects for the present study were a group of eight adult male starlings caught in the wild as adults 2 years before the experiment and kept since then in an outdoor aviary. Six months before the behavioral observations, they integrated a mixed group of 20 starlings (9 females and 3 other males) and they were observed during the non-breeding season which is favorable for determining social preferences (e.g. Henry et al.^30^). After the end of the behavioral observations, we recorded the neuronal activity of both left and right HVC during the playback of individual whistles of either familiar or unfamiliar male starlings (Fig. 1) using multielectrode systematic recordings^35^. We calculated the proportion of neuronal sites of both hemispheres responding to these individual whistles and used a hierarchical ascending classification based on an ANOVA to build individual neuronal profiles that integrate both neuronal preferences for familiar versus unfamiliar songs and asymmetries in brain processing.

## Results

**Figure 2:**
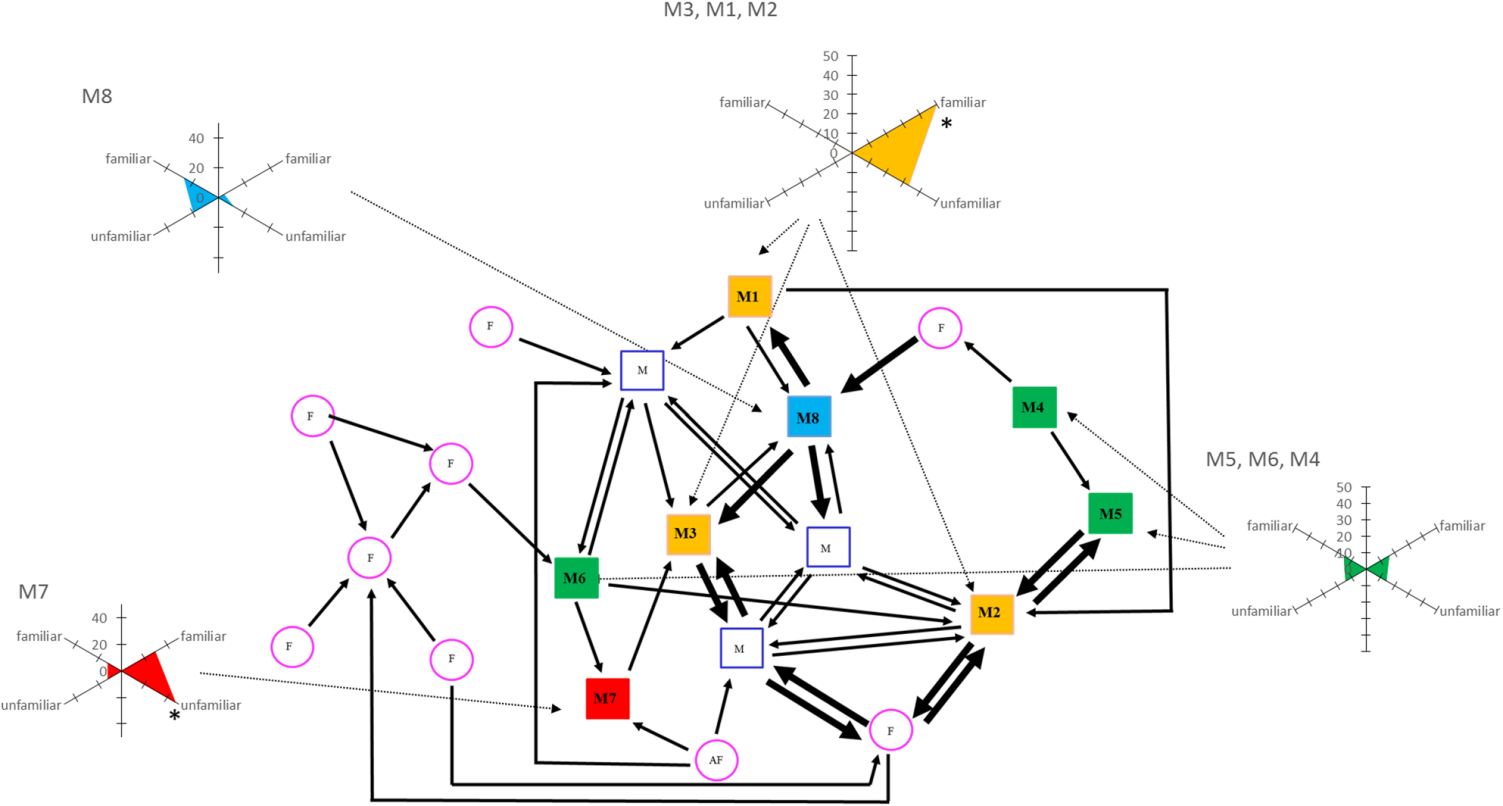
Spatial preferences and neuronal profiles of adult male starlings in our group of starlings (A2). Sociogram of proximity: Squares represent males and circles represent females. Filled colored squares represent males whose neuronal activity was recorded. Open blue squares and pink circles represent individuals that were housed in the aviary with the 8 experimental starlings. Solid black arrows link birds that were observed more often than expected by chance with another bird as their closest neighbor (χ^2^ test, large arrow: *p* ≤ .001, thin arrow, *p* ≤ .05). The different colors correspond to different clusters of neurophysiological profiles that are represented as radar plots. Each radius corresponds to the proportion of neuronal sites that responded to either the familiar or the unfamiliar stimuli in the left (left axes) and right (right axes) hemispheres.

The birds spent most of their time in close proximity to another bird (only in 16.64 ± 8.53% of the scans was a subject at more than 50 cm from another bird). Aggression was rare (only threats: 1.36 aggression/hour/individual). The sociogram showed a pattern with strong dyadic spatial preferences and some birds that were less integrated (Fig. 2).

We recorded the brain activity of 1,264 neuronal sites (632 sites/hemisphere; mean ± S.E.: 158 ± 17.28 sites/bird). Among these sites, 140 (11.1%) responded to at least one of the acoustic stimuli.

Significantly more neuronal sites were responsive in the right (mean ± S.E.: 10.87 ± 3.94) than in the left (mean ± S.E.: 6.75 ± 3.84) hemisphere (Wilcoxon test, n = 8, T = 1.5, *p* = 0.02). Each neuronal site responded to 2.70 ± 0.20 stimuli (mean ± SE). Most of them responded to only one or two stimuli (66.4%) whereas 33.5% responded to more than 2 stimuli.

Overall familiar stimuli elicited more responses than unfamiliar songs (Wilcoxon test, N = 7, T = 1, *p* = 0.05). However, one bird (A2M7) showed more responses to the unfamiliar songs.

**Figure 3:**
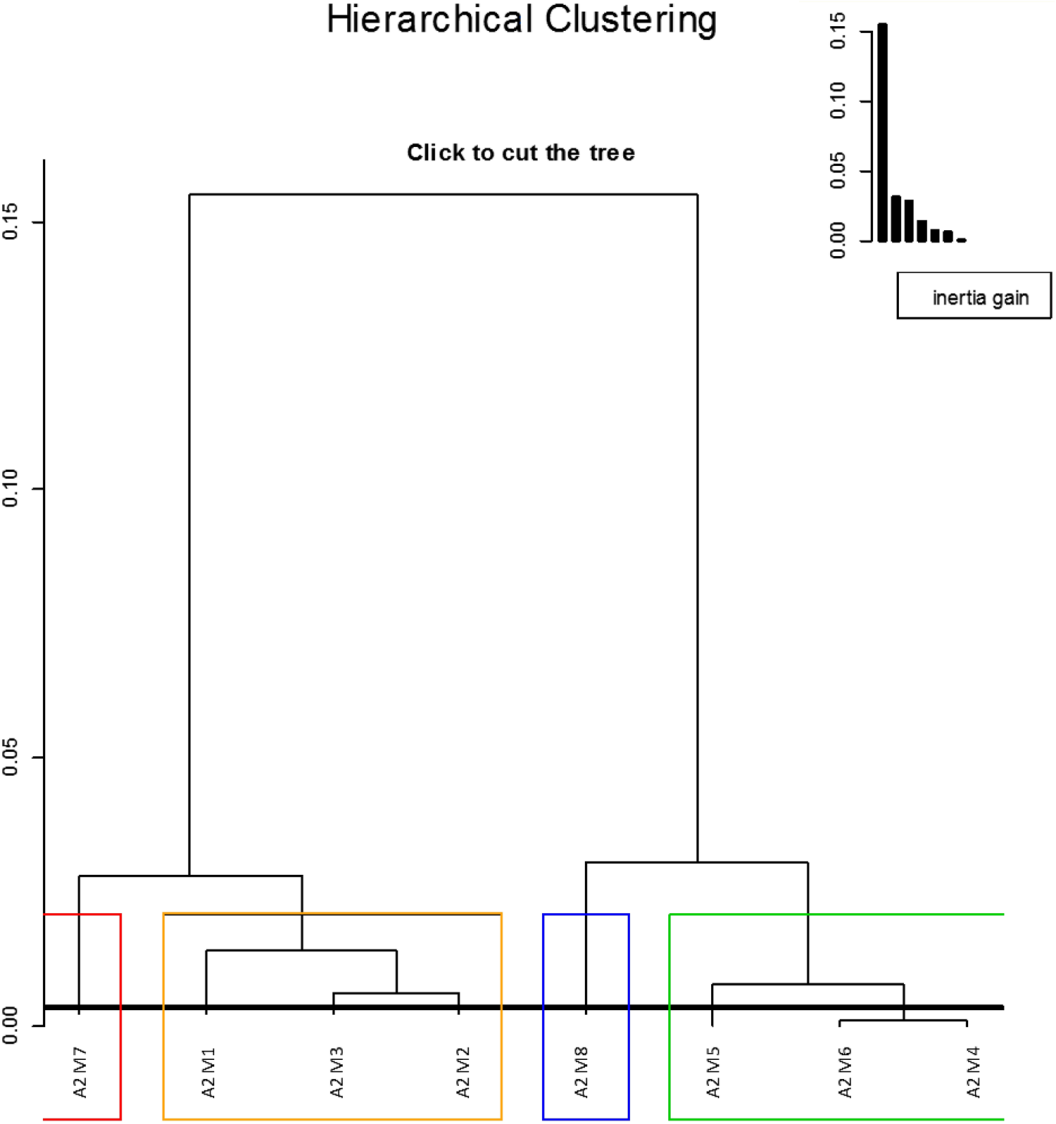
Hierarchical clustering analysis. This clustering analysis was based on a factorial analysis of responses to familiar and unfamiliar Class II whistles in the two hemispheres. The colors correspond to the clusters of birds showing the same neuronal profiles.

We then evaluated the proportions and locations (right or left) of responsive neuronal sites in relation to type and familiarity of the stimulus. A cluster analysis revealed 4 distinct individual profiles (Fig. 3): cluster 1 (one single bird: M7) was characterized by a strong right hemisphere dominance and more responses to the songs of unfamiliar than familiar birds; cluster 2 (3 birds: M1, M2 and M3 ) was characterized by a strong right hemisphere preference for familiar songs; cluster 3 (one bird: M8) was characterized by a left hemisphere dominance for both familiar and unfamiliar stimuli; and cluster 4 (3 birds: M6, M4 and M5) was characterized by bilateral responses, hence weak lateralization. The neuronal responses were thus, except for one cluster, clearly lateralized with sometimes responsive neuronal sites in one hemisphere only.

**Figure 4:**
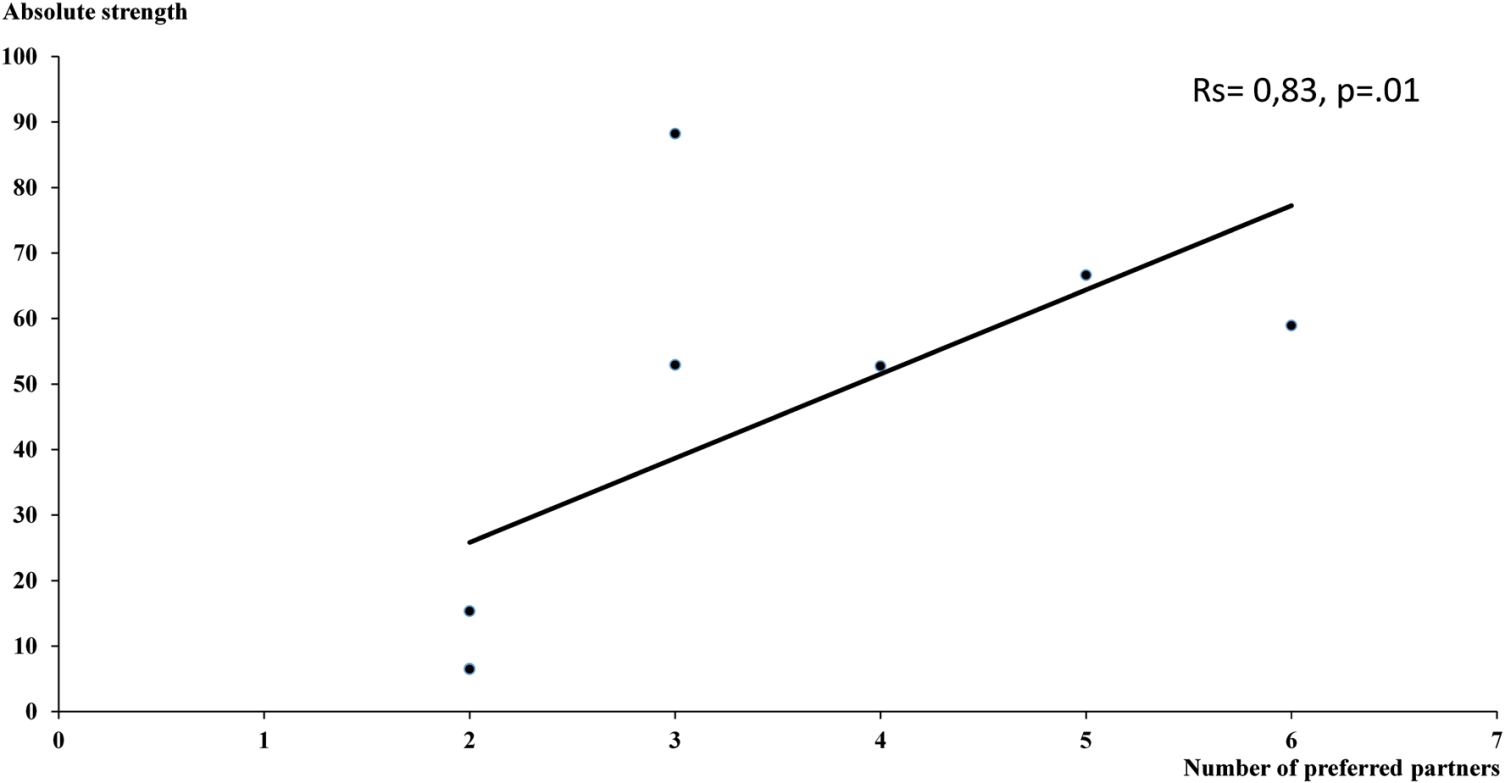
Correlation between the HVC laterality index (absolute laterality strength) and social engagement (number of significant reciprocal and non-reciprocal preferred spatial associations). (Spearman correlation test: rs = 0.83, *p* = .01).

Most importantly, we evidenced a correlation between “social engagement” (number of unilateral or bilateral associated partners) and the degree of lateralization of neuronal responses to the social acoustic signals (absolute laterality index: |L − R|/L + R; Spearman test of correlation, r = 0.83 *p* = 0.01) (Fig. 4).

The five birds (M1, M2, M3, M7 andM8) that showed neuronal responses in only one hemisphere (either the right or the left one) were involved in more preferred partnerships than the three birds that showed clear bilateral responses (M5, M6, M4) (Mann Whitney U test, n1 = 5, n2 = 3, U = 0, *p* = 0.018).

## Discussion

Our neurophysiological data show important inter-individual variations of lateralization of auditory responses. The degree of lateralization, whatever its direction, was correlated with social engagement: the closer the birds were socially associated, the more their brain processing of individual social information was lateralized. Auditory-responsive neurons in the HVC showed a bias for processing the songs of familiar rather than those of unfamiliar conspecifics and those of the most «socially engaged» subjects were clearer lateralization, with a bias in favor of the right hemisphere.

Overall, more HVC auditory-responsive neuronal sites responded to familiar songs, especially in the right hemisphere. This is in accordance with the diverse hypotheses concerning the role of this hemisphere, for example in processing individual information^36^ or attention^37^. Playback experiments showed that female starlings paid more attention to songs of familiar birds^38^, or became more aroused^39^, maybe because they were surprised to hear a familiar song in a non-familiar setting. Recent findings suggest that young mammals position themselves with their left body side towards their mother, especially in affiliative contexts, reinforcing the idea of a right hemisphere dominance for social processing^40^. Anyway, this confirms that familiarity is a major aspect of social cognition. When typical adult starlings hear a conspecific they modulate the responses of their auditory-responsive neurons according to whether the song is familiar or unfamiliar, indicating a cross-modal representation of familiar conspecifics in the brain^41^.

Lateralization of the processing of social signals is common in songbirds^42^, as in all vertebrates^14^ but its potential relationships with social integration and cohesion have not been investigated. Here we show that the more socially engaged animals presented the strongest lateralization of HVC responses to individual conspecific’s songs. The advantages of lateralization have been largely discussed, often in terms of predator avoidance (at the level of the population) or conflict resolution between the two hemispheres (at the individual level^17,43^). Recent studies related lateralization to social systems: social bee species show a lateralization pattern for diverse functions that is not found in asocial species^44^. Populational biases of the direction of lateralization have been related to sociality, hypothesizing that social coordination may require alignment^45–47^. However, asocial mason bees present the same lateralized pattern during aggressive interactions as social bees^48^. Even basic social processes may thus have triggered the evolution of lateralization^45^.

The finding here that more lateralized individuals have more preferred social partners fuels the discussion. Interestingly, the absolute degree of lateralization rather than its direction appeared important here, although overall the right hemisphere tended to present more neuronal responses. Selection for strength, but not direction, of lateralization is possible^15^; this means that strength may reflect evolutionary processes better. Since social bonding is associated with well-being^25^, being more lateralized may advantage an individual. Possibly the ability to create and maintain social bonds could be related to increased social attention, a cognitive ability that allows better vocal copying and may enhance social bonding^49,50^. Attention has been proposed as a possible important process in the evolution of lateralization^11,12^. Anyway, relationships between lateralization of social signal processing and social skills deserve further consideration thus opening future lines of research.

## Methods

### Birds

our subjects were eight adult male starlings (A2). They had been caught in the wild as adults in Normandy (France) more than 2 years before the experiment. We observed them in October while they were housed in a mixed group of 20 starlings (11 females and 1 other male) that had been together for 6 months in an outdoor aviary (2 m × 4 m × 2.5 m) with many branches, shelter and open spaces where birds could forage for insects in the soil or the air. The aviary was located in a wooded area where wild starlings could be seen and heard. Water and food were provided ad libitum (commercial pellets and apples). We ringed all birds with a unique combination of colors on both legs.

### Behavioral observations

Social preferences were evaluated following Hausberger et al.’s^29^ method. This method is based on recording the nearest neighbor (within a 50 cm radius). Social proximity is a very reliable indicator of social preferences^51,52^. We noted who was the closest neighbor using instantaneous scan sampling^53^ with one scan every 2 min. Observation sessions lasted 40 min and we obtained 323 scans per individual. For every bird, we evaluated at what frequency (proportion of scans) each other bird was its nearest neighbor. We decided whether this association occurred more or less often than expected by chance using chi-square analyses. This allowed us to include asymmetrical relationships. We then built sociograms based on spatial preferences.

We recorded agonistic interactions ad libitum because they are rare and short events.

Only one experimenter made all the behavioral observations from outside the aviary (1 m away).

### Electrophysiological recordings

In December, after the behavioral observations, we recorded the electrophysiological activity of the birds’ HVC during the playback of acoustic social signals (i.e. song elements) while they were awake and restrained. This avoided potential biases related to anesthesia, such as a loss of lateralization or selectivity in neuronal responses^12,33,34^.

Each bird was exposed to a set of stimuli of 10 Class-II individual male starling whistles (Fig. 1). These whistles characterize individual birds or closely associated birds^27^. We recorded the familiar stimuli in the birds’ home aviary. The 6 familiar whistle types differed between birds (a bird’s own songs were not included). Four stimuli came from unfamiliar distant birds, i.e. birds that the experimental animals could not have heard (e.g. recorded in New Zealand). All the recordings were made with a Marantz PMD670 digital recorder and a Sennheiser MKH416 directional microphone.

We arranged the stimuli randomly in a single sequence lasting 19.74 ± 0.02 s. The mean duration of the stimuli was 641.55 ± 27.65 ms. As HVC neurons can respond up to 350 ms after the end of a stimulus^54^, we avoided any interference between two successive responses by using a mean interval between stimuli of 722.66 ± 10.74 ms with a minimum of 514 ms.

Using the approach described by George et al.^35^, we recorded neuronal activity systematically throughout the HVC of each hemisphere while broadcasting the acoustic stimuli to starlings that were awake and restrained. We used a four-microelectrodes array (two electrodes in each hemisphere) made of tungsten wires insulated by epoxylite (FHC). Electrodes were 1.2 mm apart in the sagittal plane and 5 mm apart in the coronal plane. Electrodes’ impedance was in the range of 5–6 MΩ.

We positioned the electrodes very precisely (± 2.5 µm) using stereotaxic coordinates (first extrapolated from the canary atlas of Stokes et al.^55^ and then confirmed by the 3D atlas of the starling brain made by De Groof et al.^56^). This approach is sufficiently precise (validated by earlier histological studies^29,33^), respects the 3R rules (reducing number of animals) and keeps the animals alive.

We made 12 penetrations throughout the HVC every 230 µm in a rostrocaudal row. As we lowered the four electrodes simultaneously, it required one to three recording sessions (only one session per day) to perform all the recordings. We therefore collected data in 1–3 days. We recorded electrophysiological activity systematically every 100 µm, dorso-ventrally along the path of the penetration of an electrode without any preselection of the recording sites with acoustic test stimuli. Between the recording sessions the birds were kept in individual cages placed in the same room where the birds could hear one another and interacted vocally. Food and water were provided ad libitum.

We played the acoustic stimuli in an anechoic soundproof chamber through a loudspeaker located 20 cm in front of the bird’s head. The peak sound pressure at the bird’s ears was 85 dB SPL for all stimuli which corresponded to 65 dB RMS for all stimuli. The whole sequence of stimuli was repeated 10 times at each recording site. The neuronal activity recorded by each electrode was visualized with raster plots in which each dot represented a spike. Spikes were detected with a voltage threshold trigger. At each recording site, before playing the stimuli, the experimenter manually adjusted the amplitude discrimination to limit recordings to the neurons exhibiting the biggest spikes, with a custom-made time- and level-window discriminator^35^. The spontaneous activity of each recording site was recorded during 1.5 s before the first acoustic stimulus of each sequence.

Neuronal responsiveness was assessed by comparing spontaneous activity level (number of action potentials) with activity during stimulation using binomial tests. Only responsive sites were analyzed further. We calculated the proportion of sites responding to each stimulus and to each type of stimulus. We then used the mean values calculated for individual birds for statistical comparisons.

### Statistical analyses (see also Cousillas et al.^57^, George et al.^32^)

We used non-parametric statistics to analyze behavioral data, i.e. Chi-square tests to assess whether birds were more often than expected by chance close to some neighbors.

We compared neuronal responses between hemispheres and between stimuli using Wilcoxon tests for paired data. We compared responses to baseline with Chi-square/binomial tests.

In addition we built neurophysiological individual profiles: these profiles were built on the basis of the percentage of neuronal responses recorded in each hemisphere for each type of stimulus (familiar/unfamiliar). A hierarchical cluster analysis (HCA), based upon a Correspondence Factorial Analysis followed by a hierarchical cluster analysis was conducted to characterize individual bird’s electrophysiological profiles (Fig. 3). Birds displaying the same distribution of responses were identified and their data combined to represent profile clusters. Clusters, based on multivariate analyses, have proved useful in studies of personality and family psychology^58^ or individual behavioral profiles^50^. Each profile was represented by a radar plot and each ray of the plot represented the proportion of neuronal responses in one hemisphere for a given stimulus. Tests to identify the predominant responses on each radar were χ^2^ tests. We used Mann Whitney U tests to compare groups (i.e. clusters).

We calculated correlations between behavioral and neurophysiological data using a Spearman correlation test.

### Ethical approval

These studies comply with the French laws related to animal experimentation and the European directive 86/609/CEE and 2010/63/UE and were approved by the Rennes local Animal Care Committee (CREEA).

## Data Availability

The datasets generated during and/or analyzed during the current study are available from the corresponding author on reasonable request.
